# Single-cell Transcriptomics Uncovers the Tumor Microenvironment and Collagen-CD44 Axis in HIV Positive Cervical Squamous Cell Carcinoma

**DOI:** 10.7150/jca.134138

**Published:** 2026-07-13

**Authors:** Yuxi Ma, Lijun Zhang, Liangfei Niu, Bo Yan, Min Liu

**Affiliations:** 1 Department of Obstetrics and Gynecology, Shanghai Public Health Clinical Center, Fudan University, Shanghai 201508, China.; 2 Shanghai Public Health Clinical Center, Fudan University, Shanghai 201508, China.; 3 Center for Tuberculosis Research, Shanghai Public Health Clinical Center, Fudan University, Shanghai 201508, China.

**Keywords:** CD44, cervical squamous cell carcinoma, HIV, single-cell RNA sequencing, tumor microenvironment

## Abstract

**Introduction:**

Human immunodeficiency virus (HIV) infection markedly increases the incidence and worsens clinical outcomes of cervical squamous cell carcinoma (CSCC). The single-cell landscape of the tumor microenvironment (TME) in HIV positive CSCC (HIV-CSCC) remains poorly defined, and its cellular and molecular differences from HIV negative CSCC are largely unclear.

**Methods:**

We performed single-cell RNA sequencing (scRNA-seq) on tumor tissues from three HIV-CSCC patients without preoperative chemoradiotherapy. Public scRNA-seq data of three HIV negative CSCC cases were integrated as controls. We systematically analyzed cellular composition, transcriptional profiles, metabolic characteristics and intercellular communication networks across groups.

**Results:**

Epithelial cells in HIV-CSCC exhibited aggravated malignant phenotypes and metabolic disorders. The C5 epithelial subcluster was strongly associated with poor patient survival. Tumor-infiltrating T cells displayed impaired cytotoxic function. Macrophages predominantly polarized into immunosuppressive M2-like phenotypes with attenuated phagocytosis and antigen presentation. Cancer-associated fibroblasts (CAFs) acted as the core regulatory hub in HIV-CSCC TME and mediated cell crosstalk mainly via the collagen-CD44 signaling axis. Upregulated CD44 was an independent prognostic factor for unfavorable survival in HIV positive patients.

**Conclusions:**

HIV-CSCC is characterized by a highly immunosuppressive TME and extensive stromal remodeling driven by activated CAFs. The collagen-CD44 pathway plays a key role in tumor progression. Our findings reveal the molecular mechanism of HIV-CSCC and identify CD44 as a promising therapeutic target for this disease.

## Introduction

Human immunodeficiency virus (HIV) infection is a major risk factor for cervical cancer. Women living with HIV have approximately six-fold higher cervical cancer incidence than HIV negative individuals 1. Although antiretroviral therapy has reduced AIDS-related malignancies, cervical cancer remains one of the most prevalent cancers and a leading cause of cancer-related death in HIV positive women 2, 3. Cervical squamous cell carcinoma (CSCC) is the dominant pathological subtype, accounting for 70%-80% of all cervical cancer cases in women living with HIV. Clinically, HIV positive CSCC patients present with advanced tumor stages, rapid disease progression and inferior 5-year overall survival. Accumulating evidence demonstrates that HIV infection reshapes host immune status and tumor biological behaviors 4, 5.

HIV synergizes with human papillomavirus (HPV) to drive malignant transformation of cervical epithelial cells. HIV proteins including Tat and gp120 disrupt epithelial tight junctions and mucosal barriers 6, 7, while gp120 and Nef induce lymphocytes and macrophages to secrete pro-inflammatory cytokines 8. Increased tumor necrosis factor-α (TNF-α) and transforming growth factor-β (TGF-β) further impair intercellular junctions, forming a chronic inflammatory microenvironment that facilitates tumor development 9.

The tumor microenvironment (TME) governs tumor initiation, immune evasion, treatment response and prognosis 10. Traditional bulk RNA sequencing fails to resolve cellular heterogeneity and cell-type specific molecular signatures 11. In contrast, single-cell RNA sequencing (scRNA-seq) enables high-resolution profiling of cell subsets, transcriptional states and cell-cell communication within the TME 12. To date, no systematic single-cell study has characterized the TME of HIV-CSCC.

In the present study, we utilized scRNA-seq to map the cellular landscape of HIV-CSCC and compare it with HIV negative CSCC. This work aims to identify distinct cellular phenotypes, dysregulated signaling pathways and core communication axes, and further discover potential prognostic biomarkers and therapeutic targets for HIV-CSCC.

## Methods

### Ethical Approval

This study was approved by the Ethics Committee of Shanghai Public Health Clinical Center (Approval No.: 2024-S044-02). All experimental procedures were performed in accordance with the Declaration of Helsinki. All enrolled participants provided written informed consent, and all personal clinical information was strictly confidential.

### Patient Sample Collection

We recruited three HIV positive patients diagnosed with primary CSCC. Inclusion criteria: pathologically confirmed CSCC, HIV seropositive, no preoperative radiotherapy, chemotherapy or targeted therapy. Exclusion criteria: combined with other malignant tumors or severe systemic diseases. Three HIV negative CSCC scRNA-seq datasets were downloaded from ArrayExpress (Accession: E-MTAB-11948) and used as the control group 13. Basic clinical information of all enrolled patients is summarized in Supplementary Table 1.

### Single-Cell Suspension Preparation

Fresh tumor tissues were rinsed with ice-cold RPMI-1640 medium immediately after resection. Single-cell suspensions were prepared using Multi-tissue Dissociation Kit 2 (Miltenyi Biotec, Bergisch Gladbach, Germany) following the manufacturer's instructions. DNase I was used to reduce tissue viscosity when necessary. Red blood cells and cell debris were removed sequentially. Cell count and viability were detected by Countstar® Rigel S2 (Countstar, Shanghai, China) with AO/PI staining. Viable cells were resuspended in 1× PBS containing 0.04% bovine serum albumin (BSA) at a concentration of 1×10^6 cells/mL.

### Single-Cell RNA Library Construction and Sequencing

Single-cell transcriptome libraries were constructed using SeekOne® Digital Droplet Single Cell 3' Library Preparation Kit (SeekGene, Beijing, China). Cell suspensions and reverse transcription reagents were loaded into SeekOne® DD Chip S3. Barcoded hydrogel beads and partitioning oil were added to generate emulsion droplets. Reverse transcription was performed at 42 °C for 90 min and terminated at 85 °C for 5 min.

Complementary DNA (cDNA) was purified and amplified via PCR. Purified cDNA fragments were processed for end repair, A-tailing and adapter ligation. Indexed PCR was applied to enrich fragments with cell barcodes and unique molecular indices. Final libraries were purified with SPRI beads and quantified by quantitative PCR (KAPA Biosystems, Wilmington, MA, USA). Sequencing was performed on Illumina NovaSeq 6000 platform (Illumina, San Diego, CA, USA) with a 150 bp paired-end strategy.

### scRNA-seq Data Processing & Batch Effect Correction

Raw reads were filtered and trimmed by Fastp (v0.20.1) 14. Clean reads were processed with SeekSoul Tools to generate gene expression matrices. All downstream analyses were performed in R (v4.4.1). Standard scRNA-seq analysis was conducted using Seurat (v4.4.0) 15. Low-quality cells were excluded based on the following thresholds: gene feature number < 500 or > 7000, mitochondrial gene expression > 10%, hemoglobin gene expression > 1%. Doublets were identified and removed using Doublet-finder 16. Gene expression was normalized, and the top 2000 highly variable genes were selected. Principal component analysis (PCA) was applied for dimensionality reduction. The Harmony package (v1.2.3) was used to eliminate batch differences between internal samples and public datasets 17. Uniform manifold approximation and projection (UMAP) and t-distributed stochastic neighbor embedding (t-SNE) visualizations confirmed effective batch removal with well-mixed cells from two groups. Cell clustering was performed at a resolution of 0.5. Cell types were annotated according to canonical markers from CellMarker database and published literature 18-20. The miloR package (v1.6.0) was used to assess differential cell abundance across cell neighborhoods (50-200 cells per neighborhood) 21.

### Copy Number Variation Analysis

Large-scale copy number variations (CNVs) were inferred using InferCNV (v1.16) 22. Endothelial cells and mast cells were set as reference cell populations. Gene expression values were rescaled to -1 to 1. CNV scores were calculated as the sum of squared deviations across genomic regions.

### Pathway Enrichment Analysis

Differentially expressed genes (DEGs) identified by pseudo-bulk with *P-*value < 0.05 and |avg_log2FC| > 0.5 were subjected to Gene Ontology (GO) enrichment analysis using clusterProfiler (v4.14) 23.

### Gene Set Variation Analysis

Gene set variation analysis (GSVA) was performed based on the Molecular Signatures Database (MSigDB). Pathway activity across cell clusters and groups was visualized via pheatmap (v1.0.1) 24.

### Transcription Factor Activity Analysis

Differential transcription factor activity between groups was predicted using DecoupleR (v2.6.0).

### Pseudotime Trajectory Analysis

Cell differentiation potential was evaluated by CytoTRACE2 (v1.1.0). Cell differentiation trajectories were constructed using Monocle2 (v2.24) with the DDRTree algorithm 25. Differentiation-related DEGs were identified via differential GeneTest function.

### Cell-Cell Communication Analysis

Intercellular communication networks were predicted and compared using CellChat (v1.5.2) 26. The number and strength of ligand-receptor interactions were quantitatively analyzed.

### Confirmation of Cell Type-specific Gene Expression and Survival Analysis in the HIV+ Tumor Molecular Characterization Project (HTMCP) Database

To further validate the gene expression of specific cell type identified in our study, we obtained clinical and gene expression data from 161 samples (HIV positive, n = 70; HIV negative, n = 91) from the Genomic Data Commons data portal (https://portal.gdc.cancer.gov/). Patients were divided into high- and low-expression subgroups according to the median value of target gene signatures. Kaplan-Meier survival curves were plotted, and log-rank tests were performed using survival (v3.2-10) and survminer (v0.4.9) packages.

### Statistical Analysis

All statistical analyses were performed in R (v4.4.1). Categorical variables were compared using the Chi-square test. Continuous variables were analyzed by unpaired two-tailed Wilcoxon rank-sum test. Survival analysis was assessed with the log-rank test. A *P*-value < 0.05 was defined as statistically significant.

## Results

### Single-Cell Transcriptomic Landscape of HIV-CSCC

**Figure 1:**
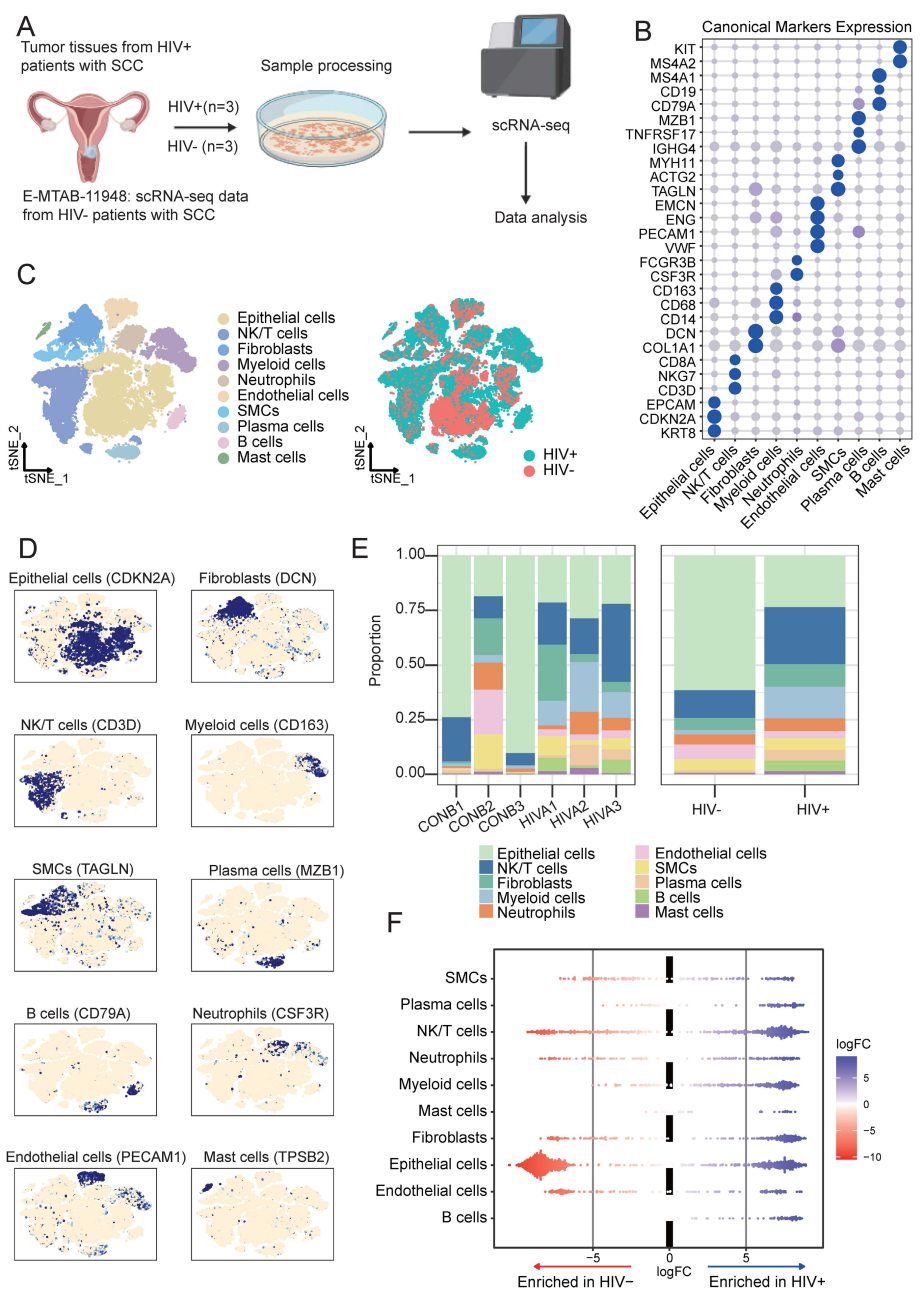
** Overview of the single-cell landscape for cervical cancer in HIV positive group and negative group. A** Workflow depicting collection and processing of specimens of tumors tissue for scRNA-seq analysis. **B** Dot plots showing the canonical markers gene expressions across ten cell types. The size of the dots represented the proportion of cells expressing the particular marker, and the spectrum of color indicated the mean expression levels of genes. **C** TSNE plots of the 46,273 cells profiled here, with each cell color coded for cell types and groups. **D** UMAP plots showing the canonical cell markers to label major cell types. **E** The proportion of all cell types between all samples and two groups. **F** Milo analysis showing the changes of cell abundance between two groups. scRNA-seq, single-cell RNA sequencing. TSNE, t-distributed stochastic neighbor embedding. UMAP, uniform manifold approximation and projection.

After strict quality control, a total of 46,273 valid cells were retained for downstream analysis (Fig. 1A), including 25,800 cells from the HIV-CSCC group and 20,473 cells from the HIV negative CSCC group. Unsupervised clustering identified 22 (C0-C21) cell clusters (Supplementary Fig. S1A and 1B), ranging from 2 cells to 5867 cells (Supplementary Table 2). Based on canonical marker expression (Fig. 1B), these cells were annotated into ten major cell populations (Fig. 1C): epithelial cells, NK/T cells, B cells, plasma cells, neutrophils, endothelial cells, myeloid cells, fibroblasts, smooth muscle cells (SMCs) and mast cells (Fig. 1D). Compared with the HIV negative group, the HIV positive group exhibited a decreased proportion of epithelial cells, accompanied by increased infiltration of NK/T cells, myeloid cells and fibroblasts (Fig. 1E). Epithelial cells were the major cells across all samples, followed by NK/T cells (Supplementary Fig. S1C). No significant differences were observed in neutrophils and SMCs between the two groups. Milo differential abundance analysis confirmed the alterations in cellular proportions shown in Fig. 1E (Fig. 1F). These results indicated that HIV infection profoundly remodeled the cellular composition of CSCC TME.

### Malignant Characteristics of Epithelial Cells in HIV-CSCC

**Figure 2:**
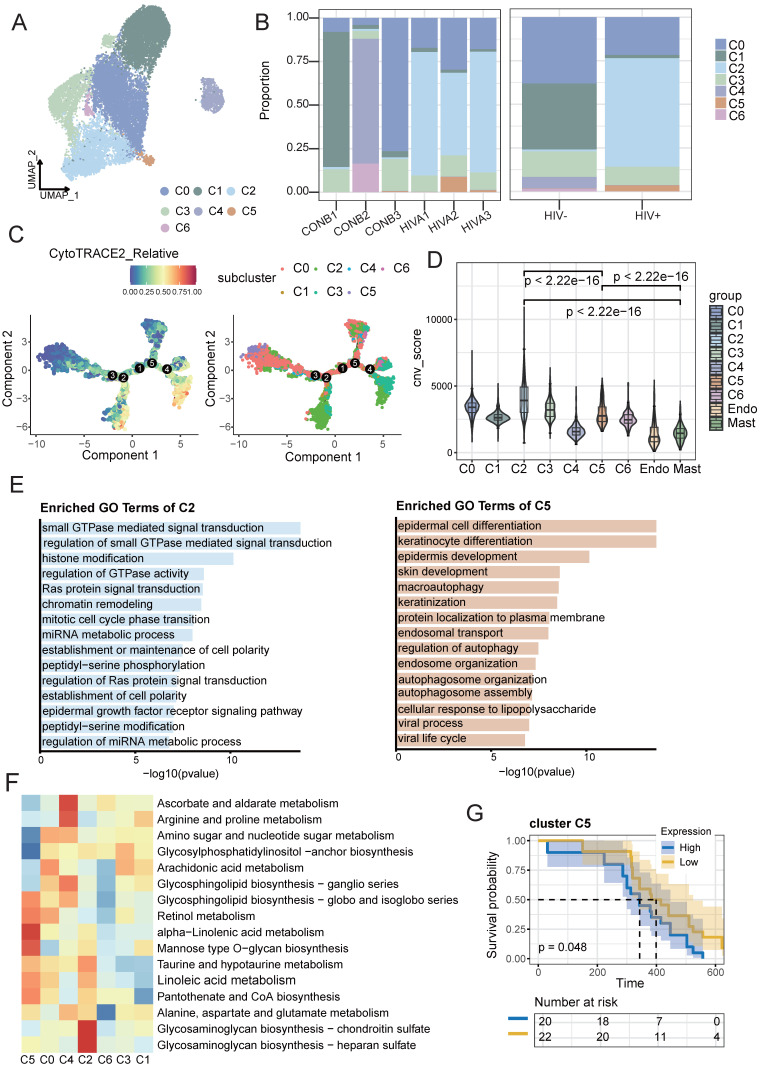
** Distinct clusters of epithelial cells in HIV positive group and negative group. A** UMAP plots of 18, 668 tumor cells profiled here, with each color coded for cell clusters. **B** The proportion of each cluster between two groups. **C** 2D graph of the pseudotime-ordered analysis of epithelial cells from two groups. **D** CNV scores of all clusters among epithelial cells. **E** GO analysis results of C2 and C5 clusters. **F** Heatmap showing the enriched metabolic pathways of epithelial cells. **G** Kaplan-Meier curve showing the overall survival rate of cervical cancer patients stratified by the top 90 genes-scaled signature of C5 cluster. UMAP, uniform manifold approximation and projection; CNV, copy number variation; GO, gene ontology.

A total of 18,668 epithelial cells were analyzed, including 6,084 cells from HIV-CSCC and 12,584 cells from HIV negative CSCC (Fig. 2A and Supplementary Fig. S2A). All epithelial cells were divided into 7 subclusters (C0-C6) (Supplementary Fig. S2B). Clusters C2 and C5 were predominantly derived from HIV-CSCC samples (Fig. 2B and Supplementary Fig. S2C). The developmental hierarchy of these clusters was examined using CytoTRACE2 and pseudotime analyses, which revealed distinct differentiation states among epithelial subclusters. C3 and C6 exhibited the highest developmental potential scores, indicative of oligopotent characteristics. C0, C1 and C2 presented unipotent features, C4 and C5 occupied terminally differentiated positions (Fig. 2C and Supplementary Fig. S2D). By calculating relative CNV scores, C2 and C5 harbored the highest CNV burden, indicating strong malignant potential (Fig. 2D).

Function enrichment analysis further highlighted distinct biological programs within these two clusters. Genes enriched in C2 was enriched in Ras-mediated signaling, establishment of cell polarity, mitotic regulation, and chromatin organization (Fig. 2E). C5 was mainly associated with epithelial differentiation and autophagy (Fig. 2E). Metabolic profiling revealed that lipid and glycerolipid metabolism were dominant in HIV-CSCC, while glycolysis was more active in HIV negative CSCC (Supplementary Fig. S2E). Glycosaminoglycan biosynthesis was enriched in C2, and α-linolenic acid metabolism was upregulated in C5 (Fig 2F).

Given their potential role in HIV-CSCC, we next evaluated the clinical relevance of cluster C2 and C5 using the HTMCP cohort. Survival analysis confirmed that the gene signature of C5 was significantly correlated with poor overall survival of HIV positive patients (Fig. 2G), whereas C2 had no prognostic value (Supplementary Fig. S2F and Table S3). These findings identified C5 as a malignant epithelial state linked to poor prognosis and suggested that it may represent a key driver of tumor progression in HIV-CSCC.

### Functional Impairment of Tumor-Infiltrating T Cells in HIV-CSCC

**Figure 3:**
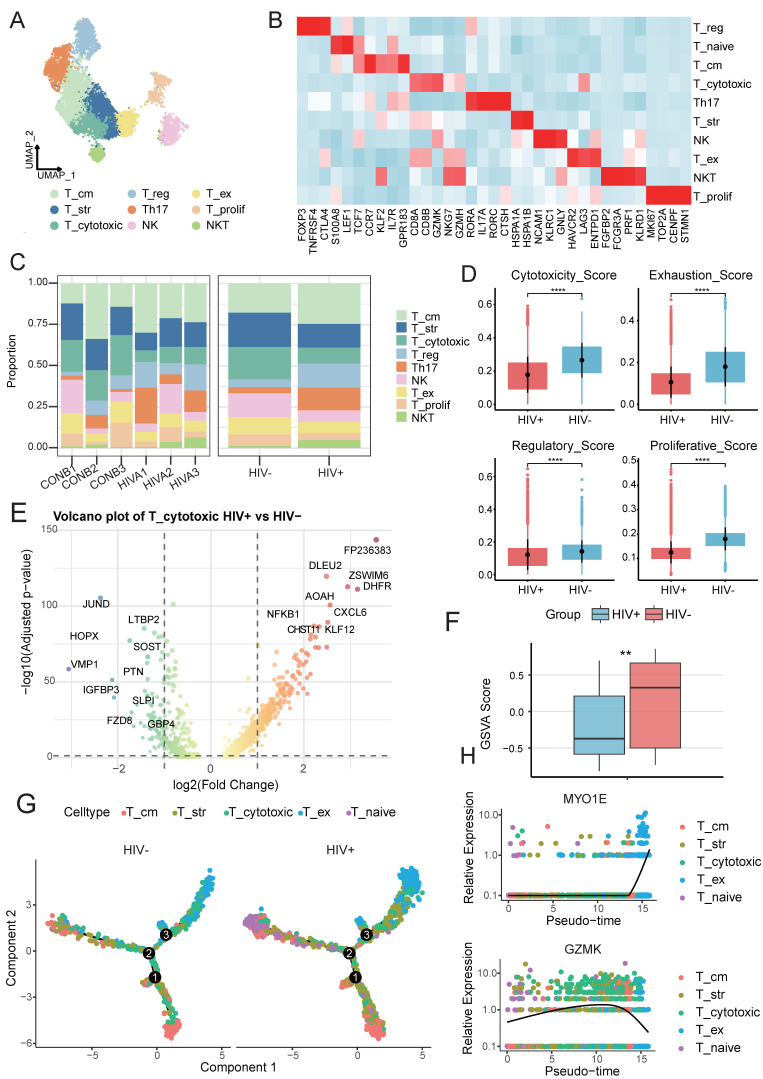
** The characteristics of NK/T clusters in two groups. A** UMAP plots of T and NK cells profiled here, with each color coded for each NK/T cell cluster. **B** Heatmap displayed the expression profiles of marker genes across NK/T cells. **C** The proportion of NK/T cells in each cluster between two groups. **D** Box plots displayed the immune scores of T cells in between two groups. ****P<0.0001. **E** Volcano plot showing the significant upregulated genes in HIV-CSCC compared to HIV negative patients. **F** Box plots displayed the cytotoxic scores between HIV positive and negative patients in HTMCP cohorts. **P<0.01. **G** 2D graph of the pseudotime-ordered analysis of T cells from two groups. **H** Dynamic changes of representative marker genes during the pseudo-time in CD8+ T cells. UMAP, uniform manifold approximation and projection. HTMCP, HIV+ Tumor Molecular Characterization Project.

Across all samples, we identified ten subclusters of NK/T cells based on canonical markers (Fig. 3A and Supplementary Fig. S3A): T_naive (LEF1, SELL), T_cytotoxic, (GZMK, NKG7), T_cm (IL7R, CCR7, GPR183), T_reg (IKZF2, IL2RA, FOXP3), T_ex (HAVCR2, LAG3), T_str (HSPA1A, HSPA1B), NK (FCER1G, NCAM1), Th17 (RORA, ADAM19, CTSH), NKT (CD8A, NKG7) and T_prolif (CENPF, MKI67, TOP2A) (Fig. 3B). HIV-CSCC group had higher proportions of Treg, Th17, and NKT cells, along but lower proportion of T_cytotoxic (Fig. 3C and Supplementary Fig. S3B). GSVA revealed different functions of each subcluster (Supplementary Fig. S3C). The distribution of these cells varied significantly across different samples, reflecting the high heterogeneity and complexity of the TME.

By calculating cytotoxicity/exhaustion/regulation/proliferation scores using GSVA (Supplementary Table 4), T cells from HIV-CSCC had significantly lower cytotoxicity, exhaustion, regulatory and proliferative activity (Fig. 3D). Downregulated genes calculated by pseudo-bulk of T_cytotoxic from HIV-CSCC enriched in biological processes linked to cytoplasmic translation, oxidative phosphorylation, and ATP synthesis via the electron transport chain pathways, suggesting sever T cells exhaustion (Fig. 3E and Supplementary Fig. S3D). Analysis of the HTMCP cohort further validated significantly lower cytotoxicity scores in HIV positive patients than in HIV negative controls (Fig. 3F), indicating severe functional impairment of CD8+ T cells in HIV-CSCC and their inability to perform effective antitumor responses. Additionally, the immune checkpoint gene CD274 (PD-L1) was upregulated, while CTLA4 was downregulated in HIV-CSCC T cells (Supplementary Fig. S3E), further confirming an immunosuppressive TME.

To investigate the differential trajectory of T cells, we constructed developmental trajectories using Monocle2. Our analysis revealed that both two groups shared similar developmental trajectories: originating from T_naive cells and progressing toward cytotoxic and exhausted states (Fig. 3G). Along the pseudotime trajectory, expression of the cytotoxic marker GZMK progressively declined, whereas exhaustion-associated gene MYO1E increased (Fig. 3H). Collectively, these findings defined HIV-CSCC as a low immune-activation state characterized by expansion of immunoregulatory T cells, depletion of cytotoxic T cells, and downregulation of effector gene expression.

### Immunosuppressive Phenotype of Myeloid Cells in HIV-CSCC

**Figure 4:**
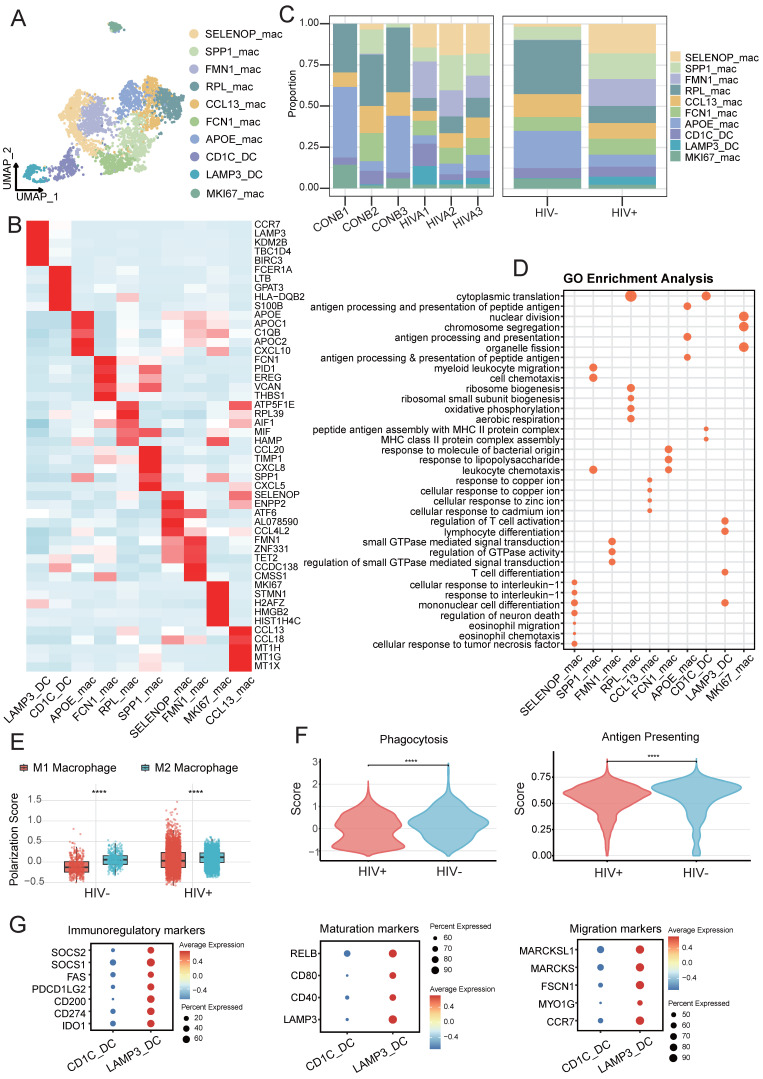
** The characteristics of myeloid cells between two groups. A** UMAP plots of 4,165 myeloid cells profiled here, with each color coded for each cell cluster. **B** Heatmap of top5 expression genes in in each identified myeloid cells clusters.** C** The proportion of myeloid cells in each cluster across all samples and groups. **D** GO analysis of each identified myeloid cells clusters. **E** Box plots showing the comparison of M1/ M2 signature scores in two groups. *P<0.05, ****P<0.0001. **F** Violin plot showing the comparison of phagocytosis and antigen presenting scores between two groups. ****P<0.0001. **G** Dot plots showing the canonical markers gene expression across DCs clusters. The size of the dots represented the proportion of cells expressing the particular marker, and the spectrum of color indicated the mean expression levels of genes. UMAP, uniform manifold approximation and projection. GO, gene ontology.

Myeloid cell infiltration is a prominent feature of many tumors and plays a central role in shaping the TME. A total of 4,165 myeloid cells were classified into eight macrophage subclusters and two dendritic cells (DCs): SELENOP_mac, RPL1_mac, FMN1_mac, SPP1_mac, CCL13_mac, FCN1_mac, APOE_mac, MKI67_mac, LAMP3_DC and CD1C_DC (Fig. 4A, B and Supplementary Fig. S4A). Macrophages represented the dominant myeloid cells and were more abundant in HIV-CSCC than in HIV negative group, SELENOP_mac specifically enriched in HIV-CSCC (Fig. 4C and Supplementary Fig. S4B) Functional enrichment analysis indicated that SELENOP_mac was strongly associated with responses to tumor necrosis factor, interleukin-1 signaling and regulation of hemopoiesis (Fig. 4D and Supplementary Fig. 4C), suggesting a role in regulating inflammatory and immune processes within the TME that may facilitate tumor progression.

Given the well-established functional polarization of macrophages, we next assessed M1- and M2-associated transcriptional programs using curated gene signatures (Supplementary Table 5) Macrophages from both groups displayed a predominantly M2-like phenotype, characterized by elevated M2 scores and relatively low M1 scores (Fig. 4E). Notably, macrophages from HIV-CSCC exhibited an even stronger M2 polarization state than those from HIV negative group. This shift was accompanied by reduced phagocytic activity and diminished antigen-presentation capacity (Fig. 4E, F).

Regarding DCs, we obtained CD1C_DC and LAMP3_DC. While CD1C_DC cells were present in both groups, LAMP3_DC cells were preferentially enriched in HIV-CSCC. LAMP3_DC cells expressed high levels of the immunoregulatory molecules IDO1 and CD274 (PD-L1) (Fig. 4G), both of which were implicated in suppressing T cells activation and function.

### Extracellular Matrix Remodeling Mediated by Cancer-Associated Fibroblasts (CAFs)

**Figure 5:**
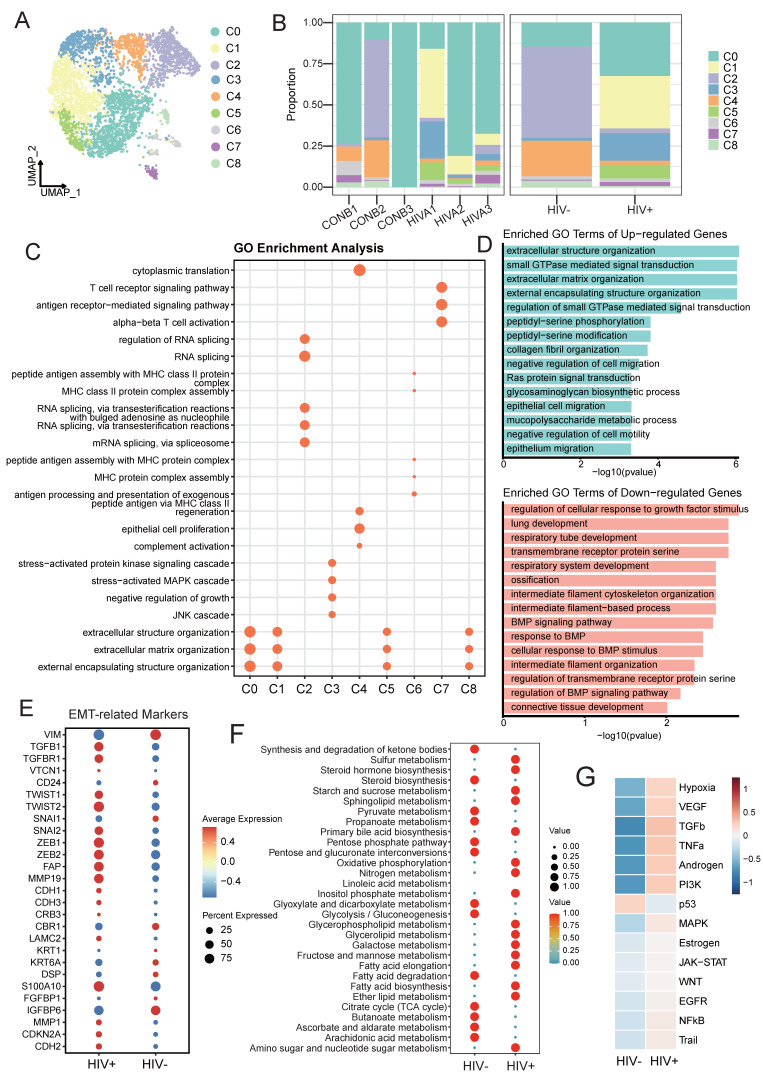
** Transcription characteristics of CAFs in HIV-CSCC and HIV negative patients. A** UMAP plots of CAFs profiled here, with each color coded for cell clusters. **B** The proportion of CAFs across all samples and groups. **C** GO analysis of CAFs clusters. **D** GO analysis of up and down regulated genes in two groups. **E** Dot plot showing the expression of EMT-related genes between two groups. **F** Bubble plot showing the enriched metabolic pathways of CAFs between two groups. **G** Heatmap showing the relative expression of differential transcription pathways between two groups. CAFs, cancer-associated fibroblasts. EMT, Epithelial-mesenchymal transition. UMAP, uniform manifold approximation and projection. GO, gene ontology.

We re-clustered 3,828 CAFs into 9 subclusters (C0-C8) (Fig. 5A, Supplementary Fig. S5A and B). We divided them into matrix CAFs (MMP1, POSTN, CTHRC1) includingC0, C1, C5, C8; inflammatory CAFs (CFD, C3, APOD) including C2, C3, C4 and C6, C7 were annotated as antigen-presenting CAFs (HLA-DRA, HLA-DRB, PTPRC). Among these, C1, C3, and C5 clusters were predominantly from HIV-CSCC, whereas C2 and C4 were mainly derived from HIV negative group (Fig. 5B and Supplementary Fig. S5C). GO functional analysis revealed that matrix CAFs were primarily associated with extracellular matrix (ECM) organization, maintenance, and structural remodeling. The inflammatory CAFs were enriched for complement activation, humoral immune responses, cytoplasmic translation, and RNA splicing-related processes. Antigen-presenting CAFs showed enrichment of pathways involved in MHC class II complex assembly and exogenous antigen processing and presentation (Fig. 5C). Upregulated genes identified by pseudo-bulk were primarily involved in epithelial cell migration, ECM synthesis and cell adhesion (Fig. 5D and Supplementary Fig. S5D). In contrast, downregulated genes primarily associated with protein synthesis and RNA processing. Key epithelial-mesenchymal transition (EMT) regulators, including ZEB1, ZEB2, SNAI2, TWIST1, TWIST2, CDKN2A, and MMP1, were significantly upregulated in the HIV positive group (Fig. 5E). Metabolic profiling further revealed enrichment of lipid and carbohydrate metabolic pathways in CAFs from HIV-CSCC (Fig. 5F and Supplementary Fig. S5E), suggesting extensive metabolic reprogramming within the stromal compartment. Transcription factor activity analysis demonstrated increased activity of multiple EMT-associated regulators, including SNAI1, NOTCH1, SMAD3, TCF3, and KLF8, in HIV-CSCC CAFs. This was accompanied by activation of key tumor-promoting pathways, including WNT, VEGF, EGFR, and TGF-β signaling (Fig. 5G and Supplementary Fig. S5F). Taken together, these results indicated that CAFs in HIV-CSCC exhibit enhanced ECM remodeling activity, suggesting a potential role in promoting an invasive TME.

### Collagen-CD44 Signaling Axis Correlates with Immunosuppression and Poor Prognosis

**Figure 6:**
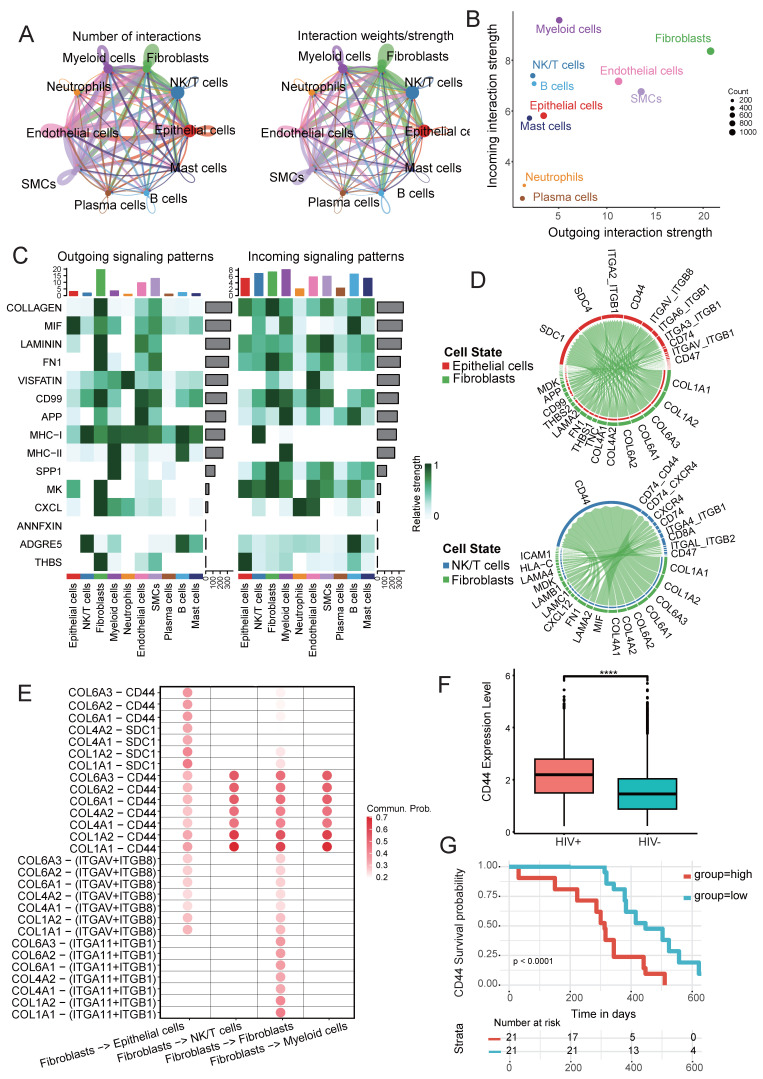
** Cellular crosstalk in HIV-CSCC microenvironment. A** Cell-cell communications between the identified cell types in HIV positive group. **B** Dotplot showing the outgoing and incoming interaction strength in HIV positive group. **C** Heatmap showing the incoming and outgoing signaling pathways of each cell type in HIV positive group. **D** Highly communicated ligand-receptor interactions between CAFs and epithelial cells and NK/T cells. **E** Dot plot revealing the interactions between CAFs and epithelial cells, NK/T cells and myeloid cells ligand-receptor pairs in cellular communication. **F** Box plots comparing the expression of CD44 in two groups. **G** Kaplan-Meier curve showing the overall survival rate of HIV positive patients in HTMCP cohorts stratified by CD44 expression. HTMCP, HIV+ Tumor Molecular Characterization Project. ****P<0.0001.

Subsequently, we performed CellChat analysis to elucidate differences in cellular communication between HIV positive and HIV negative groups. The results revealed that the total number and strength of intercellular interactions were significantly higher in HIV-CSCC (Fig. 6A and Supplementary Fig. S6A). Among these cells, CAFs emerged as the dominant communication hub, displaying extensive interactions with multiple cell types and acting as the principal source of ligand-receptor signaling within the TME (Fig. 6B and Supplementary Fig. S6B). Compared with other signaling pathways, the collagen signaling pathway represented the main signaling network in HIV-CSCC (Fig. 6C and Supplementary Fig. S6C). Given the central role of CAFs in these networks (Supplementary Fig. S6D), we next focused on CAFs-mediated interactions with epithelial cells, NK/T cells, and myeloid cells. Strong communication probabilities were observed between CAFs and epithelial cells, primarily through collagen ligands engaging receptors including syndecan-1 (SDC1), syndecan-4 (SDC4), integrin αVβ8-related complexes (ITGAV, ITGB1, and ITGB8), and CD44 (Fig. 6D). Interactions between CAFs and NK/T cells were likewise dominated by CD44 signaling, a pattern that was also evident in CAFs to myeloid cells communication (Fig. 6D and Supplementary Fig. S6E).

Notably, type I collagen emerged as the major CAFs derived ligand across all three cells, while CD44 represented a common receptor shared by epithelial cells, NK/T cells, and myeloid cells. These findings suggested that CAFs establish a collagen enriched ECM that broadly influences tumor cells and infiltrating immune cells through a common signaling framework. To further dissect CAFs centered communication, we examined signaling pathways originating from CAFs and identified interactions with high communication probability (prob > 0.2, *P* < 0.01). Among all significant pathways, the collagen-CD44 axis displayed the strongest signaling activity across the analyzed cell types. COL1A1 and COL1A2 were the predominant CAFs-derived ligands and primarily engaged CD44 as their cognate receptor (Fig. 6E), highlighting this pathway as a major mediator of stromal-tumor and stromal-immune crosstalk in HIV-CSCC. Given the prominence of CD44 within the communication network, we next evaluated its expression and clinical relevance. CD44 expression was significantly elevated in HIV positive CSCC compared with HIV negative group (*P* < 0.0001) (Fig. 6F and Supplementary Fig. S6F). Across the cellular landscape, CD44 was broadly expressed, with the highest levels detected in neutrophils (Supplementary Fig. S6G). Importantly, after adjusting for potential confounders including age and tumor stage, multivariable Cox regression analysis of the HTMCP cohort demonstrated that high CD44 expression remained independently associated with a significantly poorer survival outcome (HR = 5.25, 95% CI: 2.24-12.3, *P* ≤ 0.001). Consistently, Kaplan-Meier analysis stratified by CD44 expression level further corroborated this association (*P* < 0.05) (Fig. 6G). Together, these findings identified CAFs-driven collagen-CD44 signaling as a dominant communication axis in HIV-CSCC and implicated it as a potential mechanism linking stromal remodeling, immune regulation, and adverse clinical outcomes.

## Discussion

Persistent infection with high-risk HPV is a prerequisite for the development of cervical cancer. Among individuals living with HIV, depletion of CD4+ T cells and broader immune dysfunction compromise HPV clearance, thereby increasing the likelihood of persistent viral infection and subsequent malignant transformation 27. Clinically, HIV positive patients with cervical cancer experience poorer outcomes, including higher rates of recurrence and mortality, than their HIV negative counterparts 28, 29. Despite these disparities, current treatment recommendations remain largely comparable between the two populations. A deeper understanding of the TME in HIV-CSCC is therefore critical for the development of more effective and personalized therapeutic strategies.

We systematically characterized the cellular landscape and functional architecture of the HIV-CSCC TME using sc-RNA seq. We identified ten major cell types with distinct transcriptional identities and uncovered substantial differences in gene expression profiles, tumor-associated signaling pathways, and metabolic states across cellular compartments. These findings provide a high-resolution view of how HIV infection reshapes the cervical cancer ecosystem and offer new mechanistic insights into the interplay between viral infection, immune dysfunction, and tumor progression. More broadly, our results establish a framework for the rational development of immunotherapeutic and microenvironment-targeted strategies for HIV-CSCC.

Despite arising from the same tissue of origin, cervical tumors from HIV positive and HIV negative patients displayed marked differences in cellular states, malignant potential, and metabolic programs. Single-cell profiling revealed a more aggressive epithelial landscape in HIV-CSCC, characterized by pronounced inter-patient heterogeneity and the emergence of distinct HIV-associated malignant clusters. Among these, epithelial clusters C2 and C5 appeared to represent key tumor-promoting states. Cluster C2 exhibited features consistent with a highly malignant phenotype, including enrichment of pathways involved in cell-cycle dysregulation, DNA damage repair, chromatin remodeling, and loss of epithelial polarity. Together, these alterations are known to facilitate uncontrolled proliferation, cytoskeletal reorganization, and activation of EMT programs that drive tumor progression and dissemination 30, 31. By contrast, cluster C5 occupied a more differentiated state along the developmental trajectory and may arise from C2 during tumor evolution. This cluster was characterized by activation of autophagy-related pathways, which can support tumor growth and survival through multiple mechanisms, including the clearance of dysfunctional mitochondria and NBR1-mediated degradation of MHC-I molecules, thereby promoting immune evasion 32. These observations provide a potential mechanistic explanation for the enhanced malignant behavior observed in HIV-CSCC. Metabolic reprogramming represents another defining feature of cancer progression 33. Compared with HIV negative group, HIV-CSCC displayed significant enrichment of lipid and glycerolipid metabolic pathways, indicating substantial alterations in tumor metabolism. Notably, cluster C2 showed strong enrichment of glycosaminoglycan biosynthesis, a pathway that facilitates lipoprotein uptake and protects tumor cells from ferroptotic cell death. Consistent with this role, inhibition of glycosaminoglycan biosynthesis or degradation of cell-surface glycosaminoglycans has been shown to impair lipoprotein uptake, sensitize cancer cells to ferroptosis, and suppress tumor growth in vivo 34. In parallel, cluster C5 was characterized by enhanced α-linolenic acid metabolism, a metabolic program previously reported to be strongly associated with cervical cancer development and progression 35. Importantly, the C5 transcriptional signature was significantly associated with poor clinical outcomes in HIV positive patients. Together, these findings suggest that C2 and C5 represent biologically distinct yet functionally interconnected malignant epithelial states that contribute to tumor progression in HIV-CSCC. Their combined roles in cellular plasticity, metabolic adaptation, and immune evasion may underlie the aggressive clinical behavior of HIV-CSCC and highlight potential targets for therapeutic intervention.

The abundance and spatial distribution of T cells within tumors are fundamental determinants of antitumor immunity. In HIV-CSCC, we observed a T-cell landscape characterized by enrichment of naïve T cells and Tregs, accompanied by a relative depletion of cytotoxic T cells. This cellular composition is indicative of a poorly activated immune microenvironment with limited antitumor activity. Beyond compositional changes, T cells from HIV positive CSCC exhibited profound functional impairment, reflected by reduced cytotoxic scores and proliferative scores. These alterations likely represent a major mechanism of immune escape in HIV-CSCC. Immune checkpoint signaling may further contribute to this immunosuppressive state. The prognostic value of PD-L1 expression in patients diagnosed with invasive cervical carcinoma has been documented 36, and in our cohort, PD-L1 was significantly upregulated in HIV positive CSCC 37, 38. Collectively, these findings suggest that immune checkpoint blockade may hold particular therapeutic relevance in HIV-CSCC. Myeloid cells constitute another critical component of the TME and exert profound effects on tumor progression and immune regulation 39. In HIV-CSCC, macrophages displayed enhanced M2 polarization, reduced phagocytic capacity, and impaired antigen presentation. These features were accompanied by enrichment of SELENOP+ macrophage cluster expressing canonical M2-associated genes, including C1QA, C1QB, and APOC1. These cells engaged in immunosuppressive interactions with CD8+ T cells through the B2M-KLRD1 signaling axis, potentially attenuating cytotoxic T-cell activity 40-42, indicating the role of tumor-associated macrophages in establishing an immunosuppressive microenvironment and promoting tumor progression. A further distinguishing feature of the TME in HIV-CSCC was the enrichment of LAMP3+ DCs, which exhibited high expression of PD-L1 and IDO1. Their impaired antigen presentation and interactions with tumor cells via VEGF/SEMA pathways likely contributed to immune evasion and angiogenesis 43, 44, raising the possibility that LAMP3+ DCs contribute not only to immune suppression but also to metastatic dissemination. Together, these observations define a profoundly dysfunctional immune landscape in HIV-CSCC, characterized by impaired cytotoxic immunity, suppressive myeloid clusters that collectively facilitate tumor progression.

Among all stromal cells, CAFs emerged as the principal source of ECM-related genes and were substantially enriched in HIV-CSCC compared with HIV negative group. Through ECM remodeling and secretion of diverse signaling molecules, these cells actively shape the TME, creating conditions that support tumor growth, invasion, and immune evasion 45. Consistent with this role, CellChat analysis identified CAFs as the dominant communication hub within the HIV-CSCC microenvironment, with collagen signaling representing the most prominent intercellular communication network. A particularly striking finding was the prominence of the collagen-CD44 signaling axis. This pathway mediated extensive interactions between CAFs and diversity cells and appeared to represent a major route through which stromal cells influence immune and tumor cell behavior. Beyond its role in cell adhesion, collagen-CD44 signaling has been implicated in directing immune cell migration and regulating cellular responses within the TME. Similar signaling networks have been reported in gastric cancer and several other malignancies 46, 47. Notably, in HIV-associated esophageal squamous cell carcinoma, CAFs-derived COL1A2 interacts with CD44-positive epithelial cells, leading to upregulation of PIK3R1 and activation of the PI3K-AKT pathway, thereby promoting tumor progression 48.

These observations suggest that collagen-CD44 signaling may represent a conserved mechanism through which stromal remodeling drives malignant progression in HIV-associated cancers. Extensive ECM remodeling has also been shown to impair antitumor immunity by restricting the access of cytotoxic immune cells to tumor nests and redirecting them toward stromal regions, ultimately facilitating immune escape 49. In this context, CD44 appears to occupy a central position within the HIV-CSCC signaling network. Beyond serving as a receptor for collagen, CD44 contributes to tumor progression through multiple mechanisms, including regulation of immune suppression, cellular migration, and tumor cell survival. Consistent with our findings, elevated CD44 expression has been associated with poor clinical outcomes in both gastric cancer and glioma patients 47, 50. Wang et al revealed that Hyaluronan-CD44 signaling represents a critical link between ECM remodeling and immune evasion in gastric cancer peritoneal metastasis. Targeting ECM-driven immunosuppressive mechanisms may represent a promising strategy to overcome therapeutic resistance and enhance the efficacy of immunotherapy in this aggressive disease 51. Moreover, it was identified that the RNA aptamer sTN58 specifically binds to CD44 on the surface of drug-resistant triple-negative breast cancer cells, blocks downstream CD44 signaling, inhibits the EMT process, and partially reverses the stem-like phenotype, thereby attenuating tumor drug resistance and invasiveness 52. Taken together, these findings position CD44 as a central signaling node that integrates stromal remodeling, immune regulation, and tumor-promoting pathways. Its broad involvement in multiple pro-tumorigenic processes highlights CD44 as a promising therapeutic target and a potential avenue for the development of novel treatment strategies in HIV-CSCC.

We acknowledge that this study has several limitations that should be considered when interpreting the results. Firstly, the relatively small sample size in our sc-RNA seq experiments may introduce sampling bias, potentially compromising the representativeness and reproducibility of the findings. Secondly, though harmony effectively mitigated the batch effects, we acknowledge that subtle platform-specific biases, particularly for lowly expressed genes cannot be entirely ruled out. Lastly, functional validation of the cluster C5 in epithelial cells was not performed. Although our analyses suggest that collagen-CD44 may play an important role in HIV-CSCC, these findings remain inferential and requires further experimental validation in large cohort studies.

## Conclusions

In summary, HIV-CSCC is characterized by a profoundly immunosuppressive TME and extensive stromal remodeling driven by activated CAFs. Malignant epithelial cells, functionally impaired immune cells and highly activated CAFs synergistically promote tumor progression. The CAF-mediated collagen-CD44 signaling axis acts as a core pathway linking ECM remodeling, immune suppression and poor clinical outcomes. This single-cell transcriptomic study elucidates the molecular pathogenesis of HIV-CSCC and highlights CD44 as a novel and potential therapeutic target for this disease.

## Supplementary Material

Supplementary figures.

Supplementary tables.

## Data Availability

The raw sequence data reported in this study have been deposited in the Genome Sequence Archive (Genomics, Proteomics & Bioinformatics 2025) in National Genomics Data Center, China National Center for Bioinformation/Beijing Institute of Genomics, Chinese Academy of Sciences (GSA-Human: HRA017151) that are publicly accessible at https://ngdc.cncb.ac.cn/gsa-human. The scRNA-seq data of HIV negative patients for this study come from the ArrayExpress database (https://www.ebi.ac.uk/biostudies/arrayexpress). The accession number is E-MTAB-11948. The bulk data were obtained from the UCSC Xena (https:// xena. ucsc. edu/). All the data in this study support the results of this study.

## Abbreviations

AIDS: Acquired Immunodeficiency Syndrome
CAFs: Cancer-associated fibroblasts
CNVs: Copy number variations
CSCC: Cervical squamous cell carcinoma
DCs: Dendritic cells
ECM: Extracellular matrix
EMT: Epithelial-mesenchymal transition
GO: Gene Ontology
GSVA: Gene set variation analysis
HIV: Human immunodeficiency virus
HPV: Human papillomavirus
HTMCP: HIV+ Tumor Molecular Characterization Project
MSigDB: Molecular Signatures Database
PCR: Polymerase chain reaction
scRNA-seq: Single-cell RNA sequencing
TF: Transcription factor
TME: Tumor microenvironment
TSNE: t-distributed stochastic neighbor embedding
UMAP: Uniform manifold approximation and projection
