# Supplementary material for: Single-cell Transcriptomics Uncovers the Tumor Microenvironment and Collagen-CD44 Axis in HIV Positive Cervical Squamous Cell Carcinoma
Source: J Cancer. 2026 Jul 13;17(7):1346–61. doi: 10.7150/jca.134138 (PMC13410425; doi:10.7150/jca.134138)
Supplement: Supplementary file 1 — Supplementary figures. [file jcav17p1346s1.pdf]

Supplemental figures

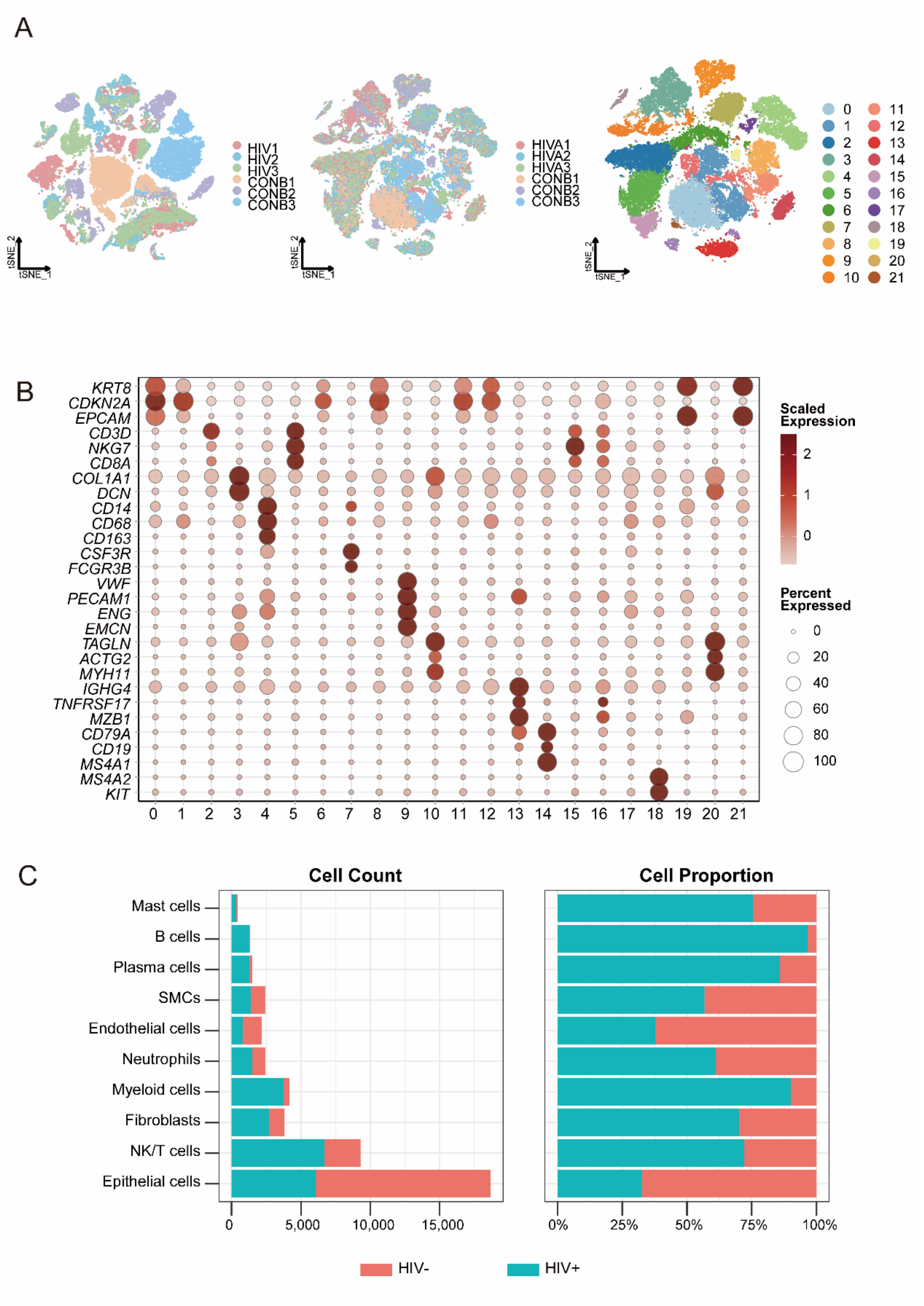

Fig. S1 Overview of single-cell clusters and cell types composition in HIV positive and HIV negative CSCC. A TSNE plots of all cells from six samples, with each cell color coded for each cluster and sample. The left plot showing all cells before harmony,

the middle and right plots showing all cells after harmony. **B** Dot plots showing the common markers gene expressions across the 22 clusters. The size of dots represents the proportion of cells expressing the particular marker, and the spectrum of color indicates the mean expression levels of the markers. **C** The proportion of all cell types between two groups.

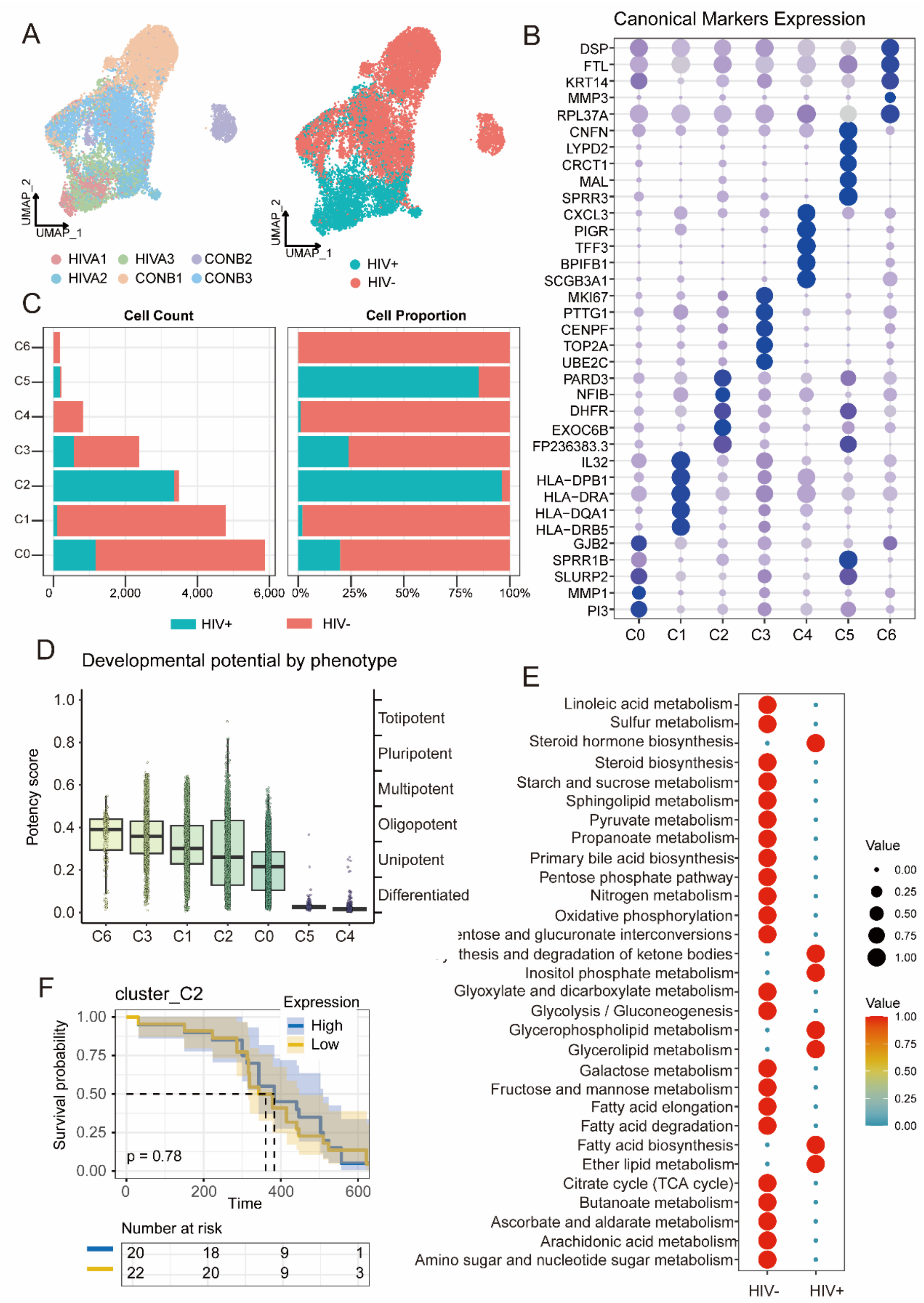

Fig. S2 Molecular and malignant characteristics of epithelial subclusters in CSCC. **A** UMAP plots of epithelial cells from six samples, with each cell color coded for each sample and group. **B** Dot plots showing the common markers gene expressions across the six clusters. The size of dots represents the proportion of cells expressing the

particular marker, and the spectrum of color indicates the mean expression levels of the markers. **C** The proportion of each cluster in epithelial cells between two groups. **D** Boxplot showing the differentiation potential categorized by phenotype. **E** Dot plot showing the enriched metabolic pathways of epithelial cells between two groups. **F** Kaplan–Meier curve showing the overall survival rate of cervical cancer patients stratified by the top 90 genes- scaled signature of C2 cluster.

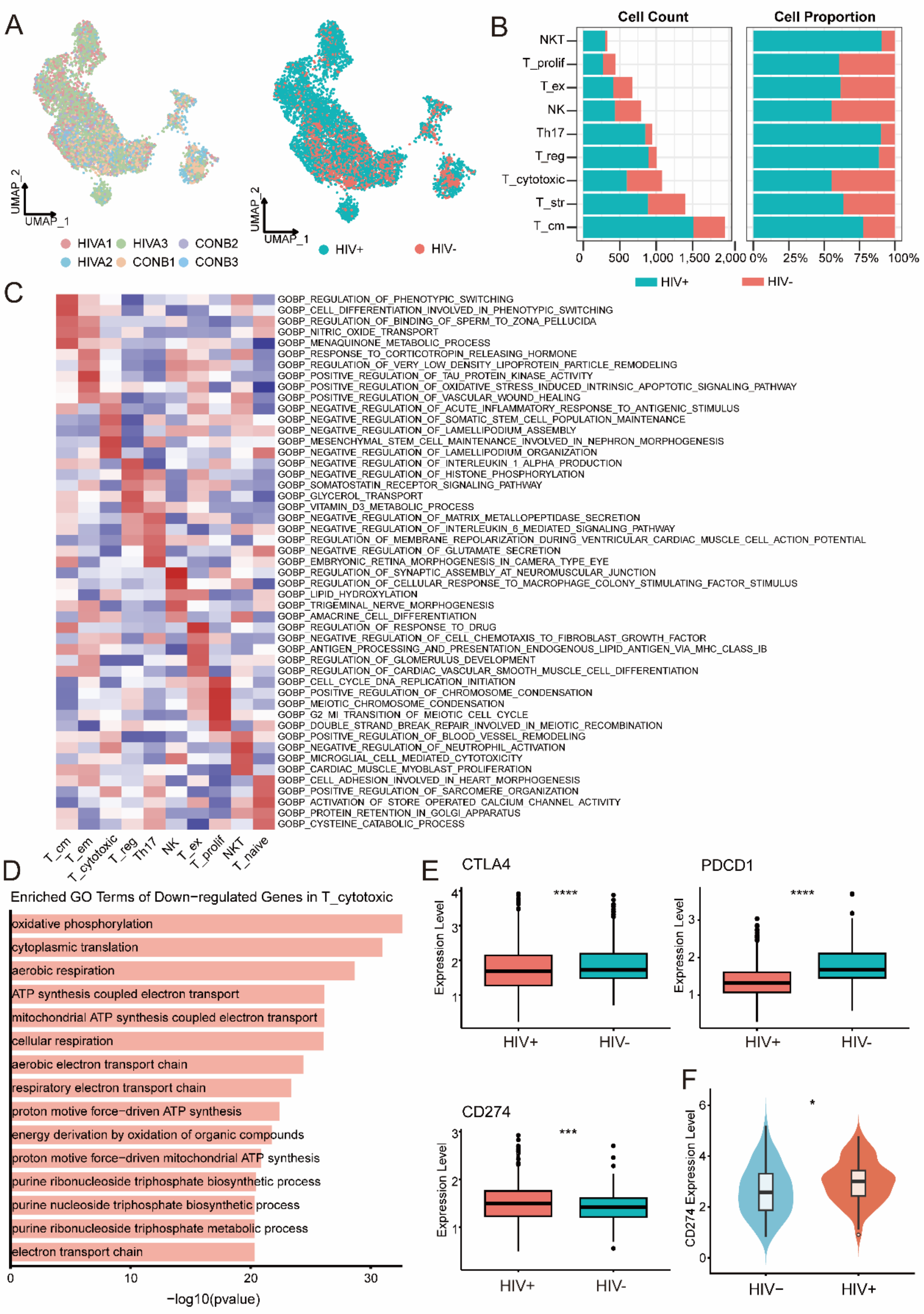

Fig. S3 Transcriptional profiles and functional characteristics of NK/T cells. A UMAP plots of NK/T cells from six samples, with each cell color coded for each sample and group. **B** The proportion of each cluster in NK/T cells between two groups. **C** Pheatmap showing the functional enrichment with GSVA in each cell type. **D** GO analysis results of down-regulated genes in T_cytotoxic. **E** Box plots showing the

expression of immune checkpoint genes in HIV positive and negative patients. **F** Violin plots confirming CD274 expression of in HTMCP cohorts. *P<0.05, **P<0.01, ***P<0.001, ****P<0.0001.

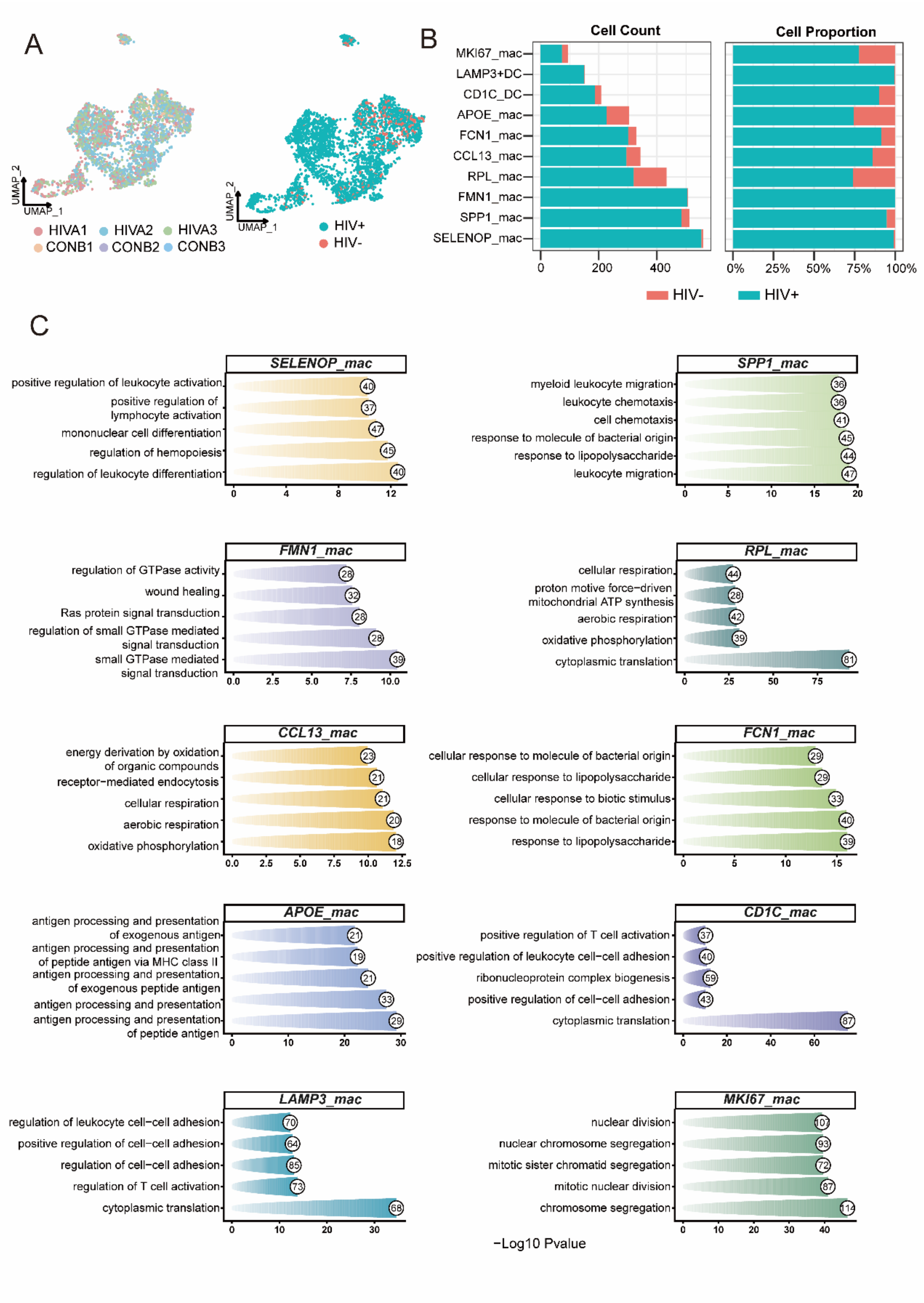

Fig. S4 Transcriptional profiles and functional characteristics of myeloid cells. A UMAP plots of myeloid cells from six samples, with each cell color coded for each sample and group. **B** The proportion of each cluster in myeloid cells between two groups. **C** Representative enriched GO terms enriched in each cell type are depicted. The numbers at the front end of the column represent the number of genes enriched in the GO term.

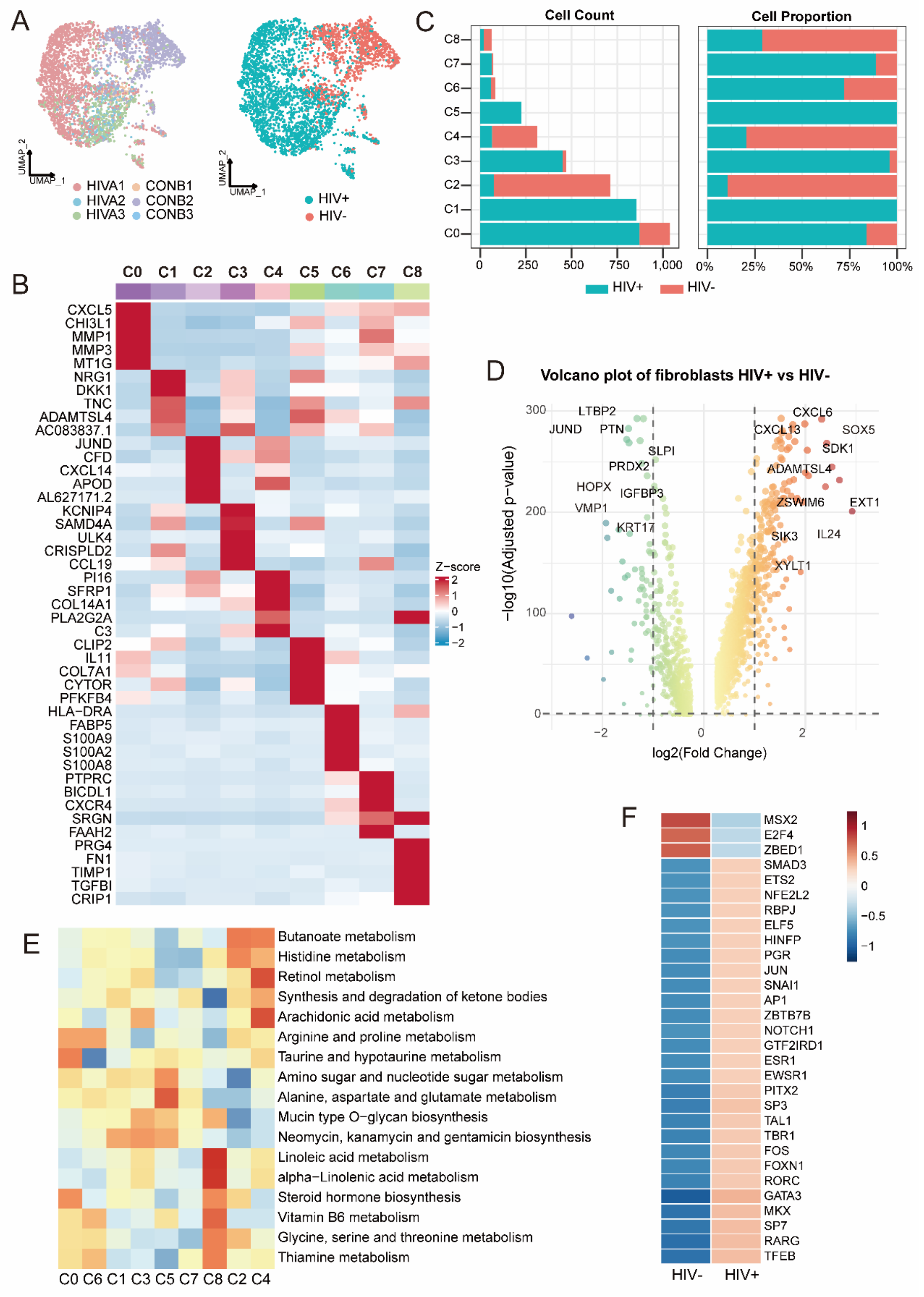

Fig. S5 Transcriptional profiles and functional characteristics of CAFs. A UMAP plots of CAFs from six samples, with each cell color coded for each sample and group. **B** Heatmap of top5 expression genes in each identified CAFs. **C** The proportion of CAFs in each cluster between two groups. **D** Volcano plot showing the significant

upregulated genes of CAFs in HIV positive group compared to HIV negative group. **E** Heatmap showing the enriched metabolic pathways of CAFs. **F** Heatmap showing the relative expression of differential transcription factors between two groups.

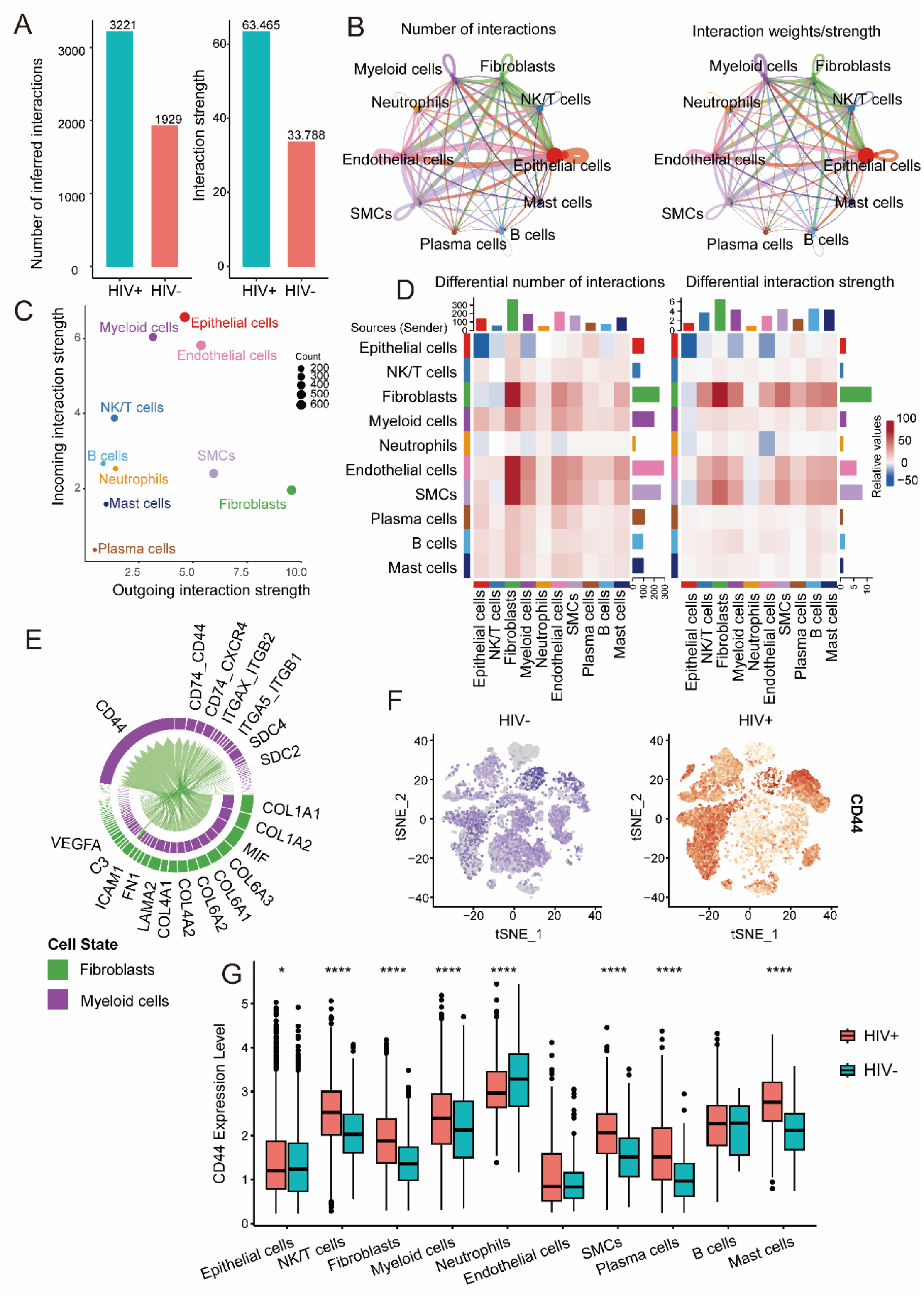

Fig. S6 Cellular crosstalk in HIV-CSCC TME. A Differences in cell communication

between HIV positive and negative patients. **B** Cell–cell communications between the identified cell types in HIV negative group. **C** Dotplot showing the outgoing and incoming interaction strength in HIV negative group. **D** Heatmap showing the number and strength of differential cell interaction in HIV positive group compared to negative group. **E** Highly communicated ligand-receptor interactions between CAFs and myeloid cells. **F** UMAP plots showing the distribution of CD44 expression levels in two groups, with color intensity positively correlated with CD44 expression levels. **G** Box plots comparing the expression of CD4 in different cell types between two groups. $^{*}P<0.05$, $^{****}P<0.0001$.
